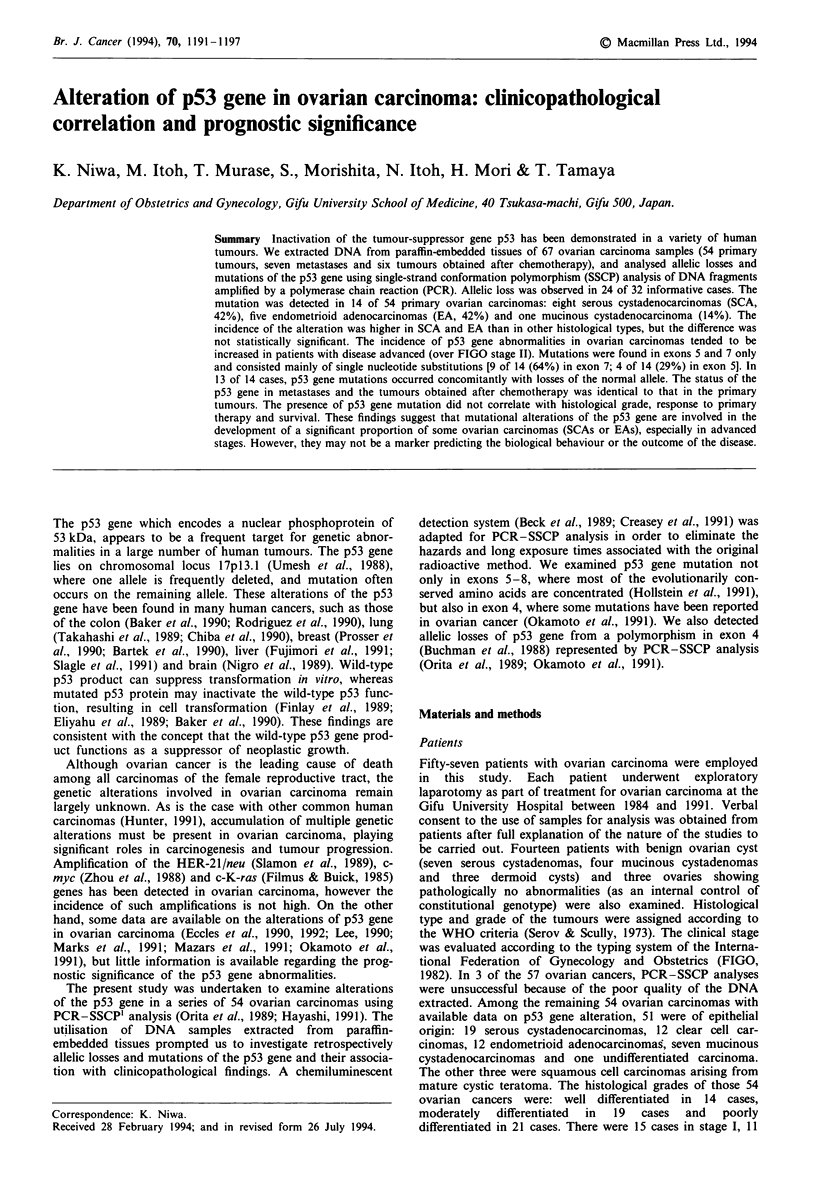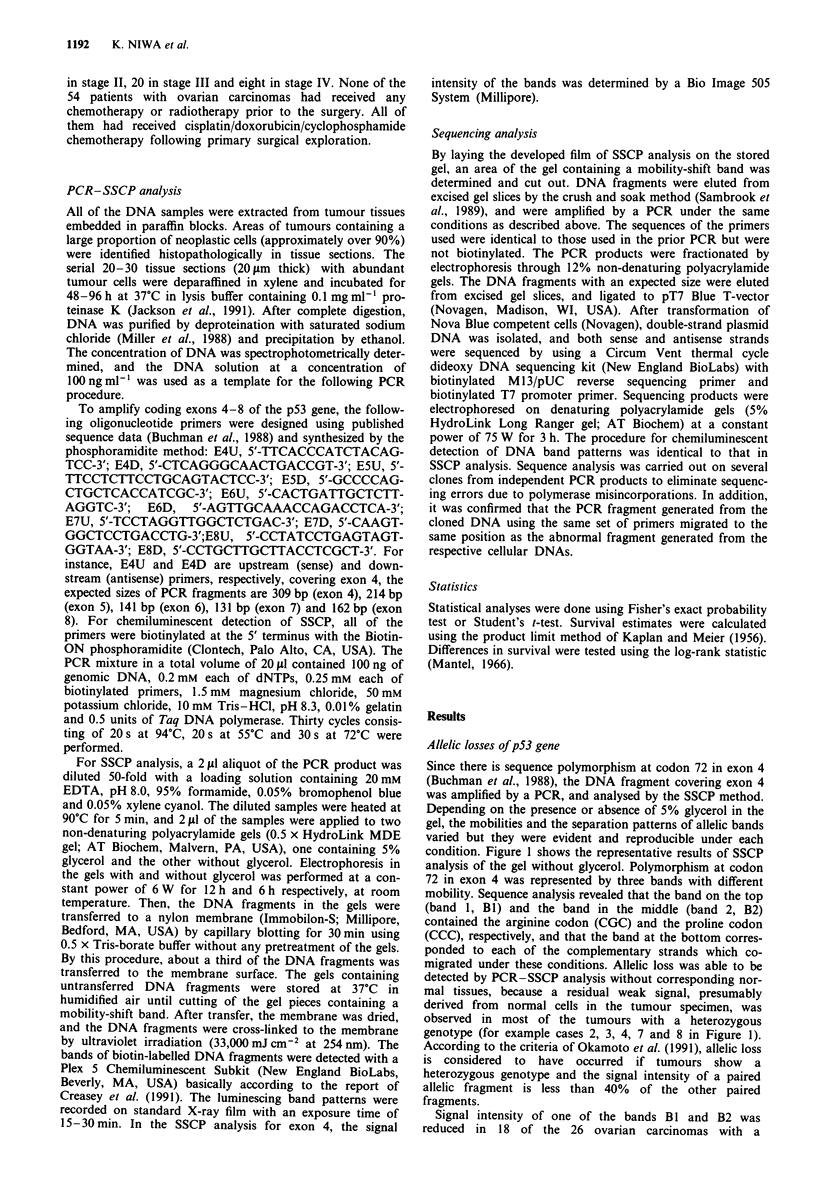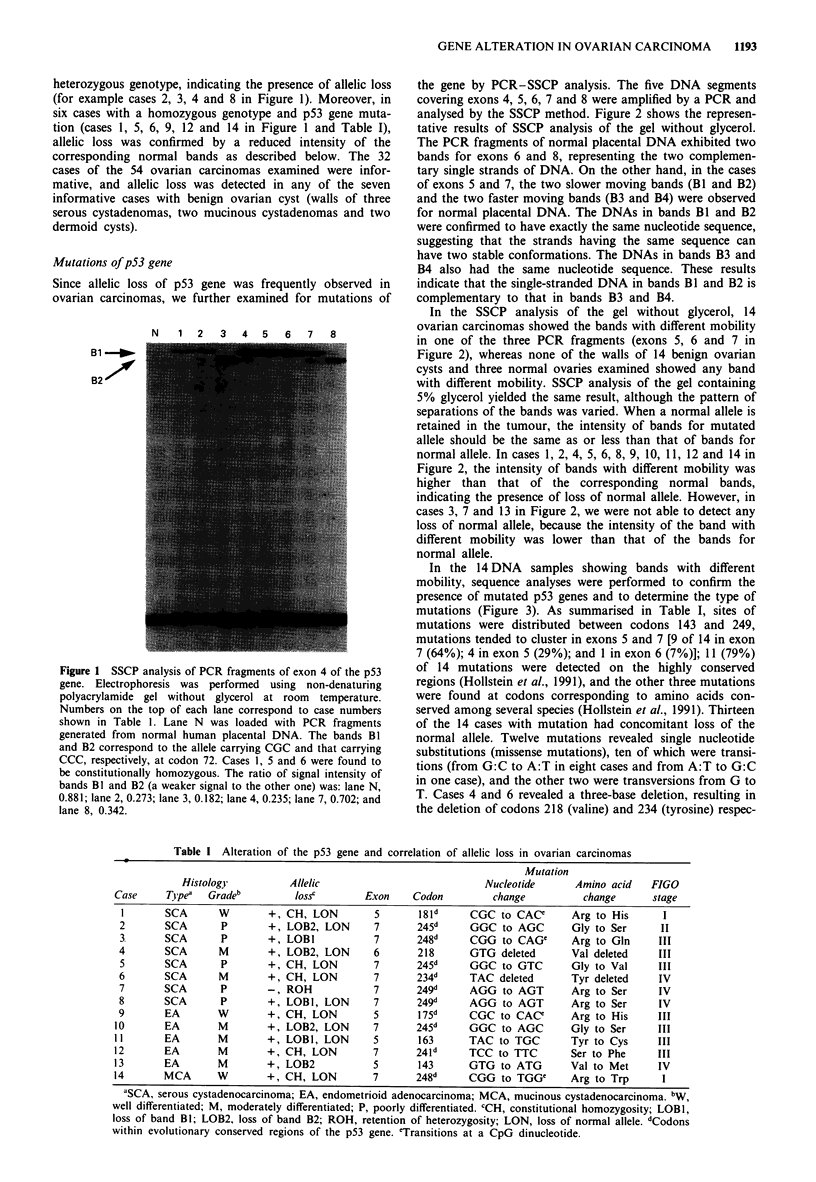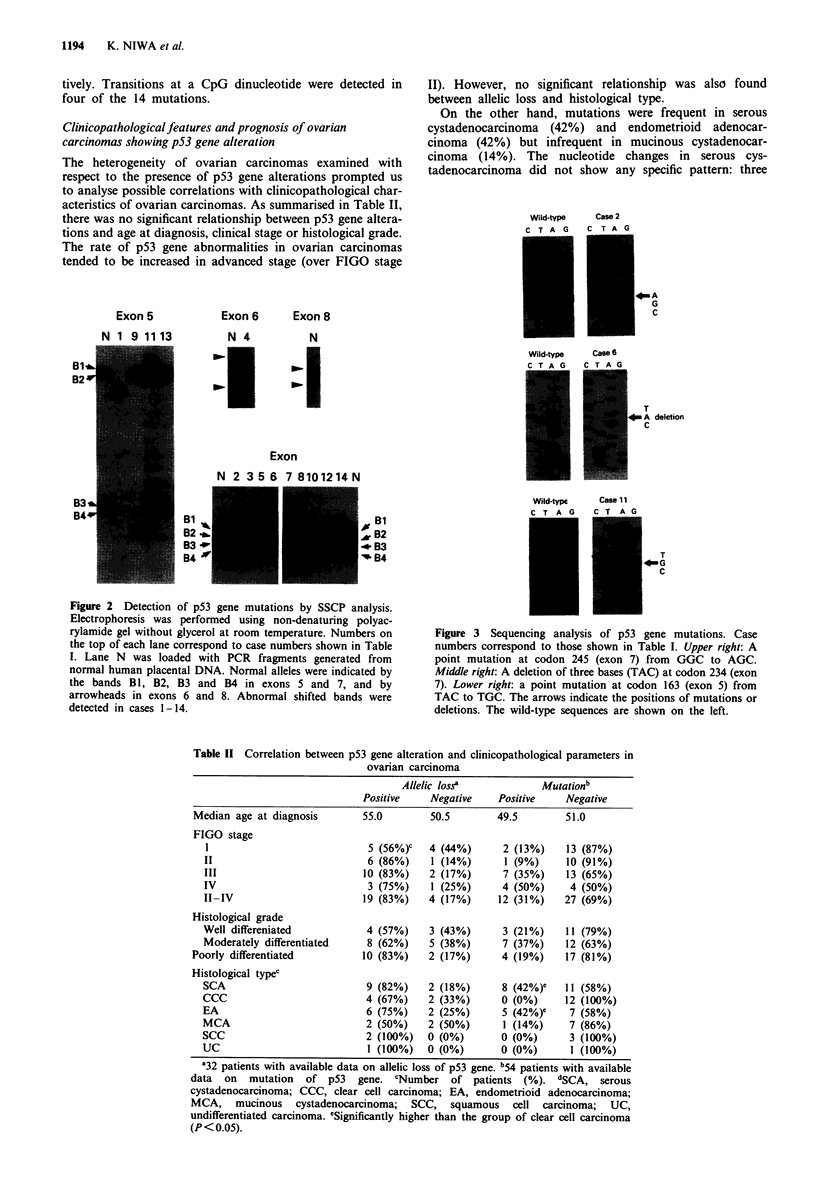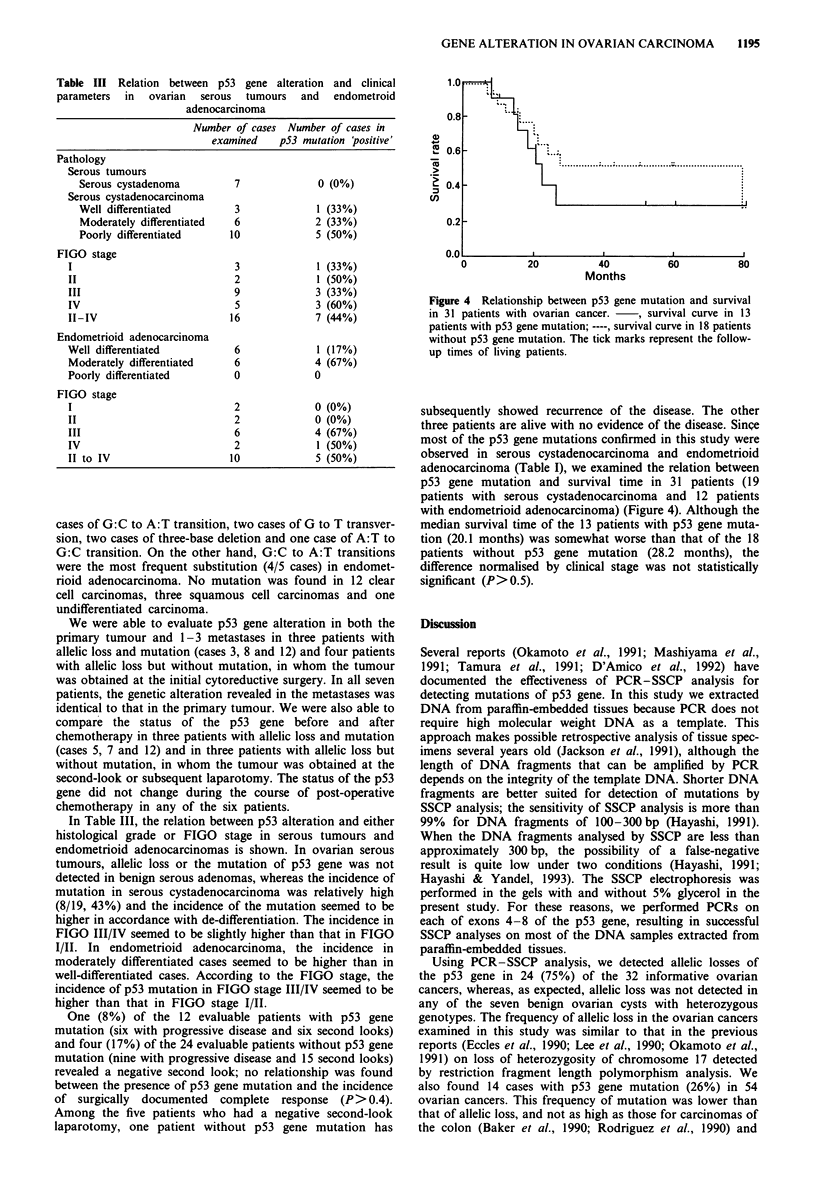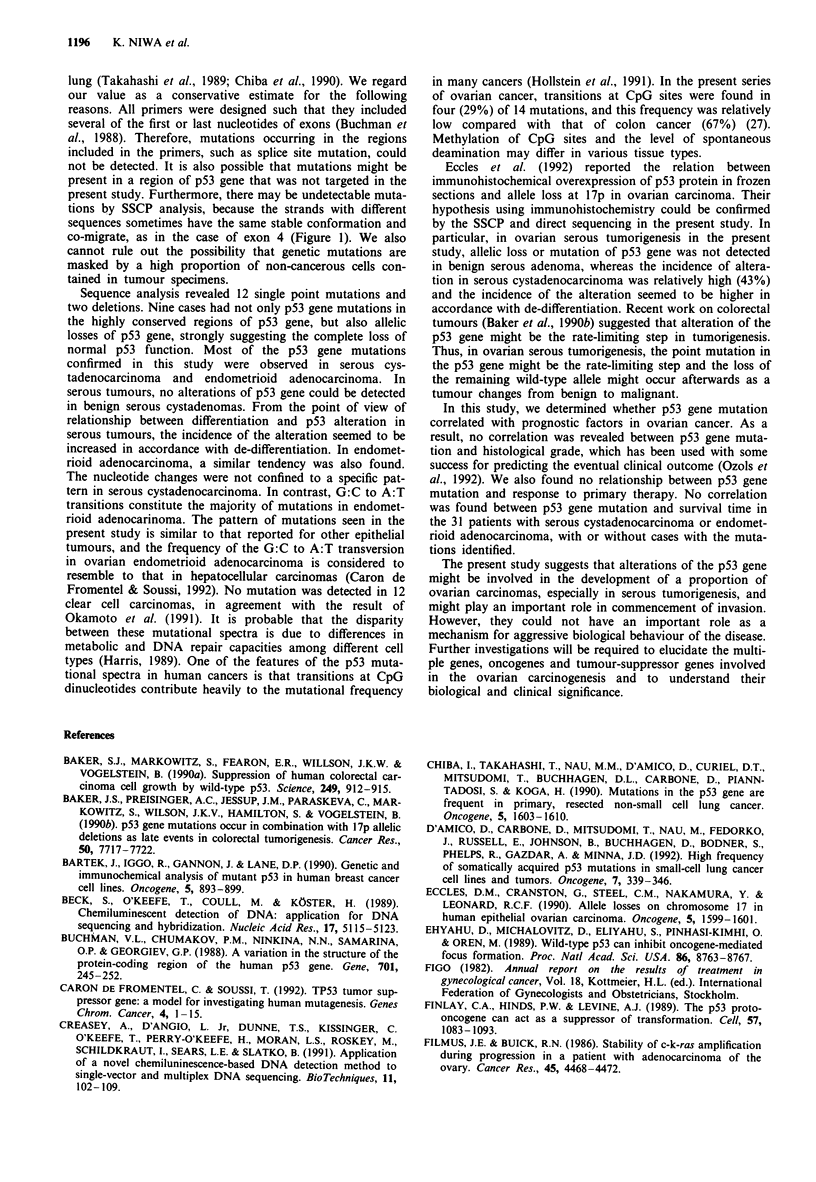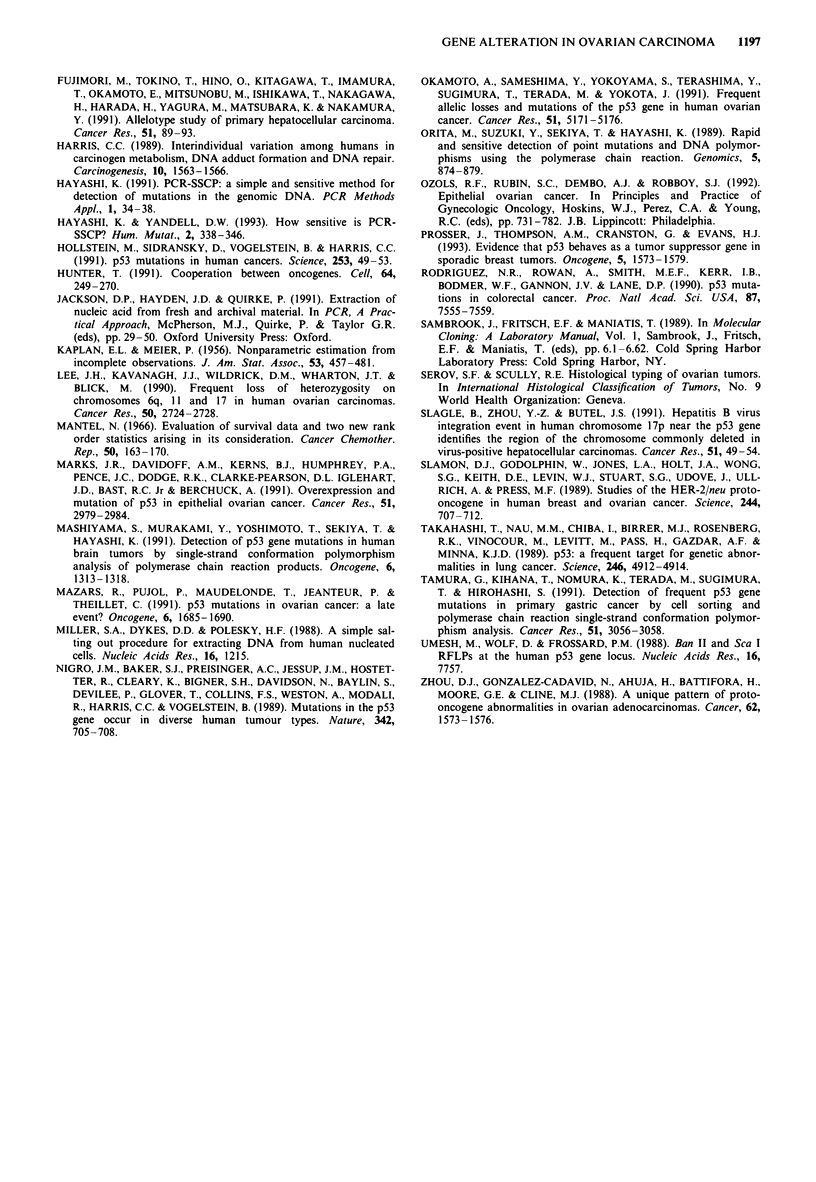# Alteration of p53 gene in ovarian carcinoma: clinicopathological correlation and prognostic significance.

**DOI:** 10.1038/bjc.1994.472

**Published:** 1994-12

**Authors:** K. Niwa, M. Itoh, T. Murase, S. Morishita, N. Itoh, H. Mori, T. Tamaya

**Affiliations:** Department of Obstetrics and Gynecology, Gifu University School of Medicine, Japan.

## Abstract

**Images:**


Br. J. Cancer (1994), 70, 1191-1197 © Macmillan Press Ltd., 1994

### Alteration of p53 gene in ovarian carcinoma: clinicopathological correlation and prognostic significance

K. Niwa, M. Itoh, T. Murase, S., Morishita, N. Itoh, H. Mori & T. Tamaya

*Department of Obstetrics and Gynecology, Gifu University School of Medicine, 40 Tsukasa-machi, Gifu 500, Japan.*

**Summary** Inactivation of the tumour-suppressor gene p53 has been demonstrated in a variety of human tumours. We extracted DNA from paraffin-embedded tissues of 67 ovarian carcinoma samples (54 primary tumours, seven metastases and six tumours obtained after chemotherapy), and analysed allelic losses and mutations of the p53 gene using single-strand conformation polymorphism (SSCP) analysis of DNA fragments amplified by a polymerase chain reaction (PCR). Allelic loss was observed in 24 of 32 informative cases. The mutation was detected in 14 of 54 primary ovarian carcinomas: eight serous cystadenocarcinomas (SCA, 42%), five endometrioid adenocarcinomas (EA, 42%) and one mucinous cystadenocarcinoma (14%). The incidence of the alteration was higher in SCA and EA than in other histological types, but the difference was not statistically significant. The incidence of p53 gene abnormalities in ovarian carcinomas tended to be increased in patients with disease advanced (over FIGO stage II). Mutations were found in exons 5 and 7 only and consisted mainly of single nucleotide substitutions [9 of 14 (64%) in exon 7; 4 of 14 (29%) in exon 5]. In 13 of 14 cases, p53 gene mutations occurred concomitantly with losses of the normal allele. The status of the p53 gene in metastases and the tumours obtained after chemotherapy was identical to that in the primary tumours. The presence of p53 gene mutation did not correlate with histological grade, response to primary therapy and survival. These findings suggest that mutational alterations of the p53 gene are involved in the development of a significant proportion of some ovarian carcinomas (SCAs or EAs), especially in advanced stages. However, they may not be a marker predicting the biological behaviour or the outcome of the disease.

The p53 gene which encodes a nuclear phosphoprotein of 53 kDa, appears to be a frequent target for genetic abnormalities in a large number of human tumours. The p53 gene lies on chromosomal locus 17p13.1 (Umesh *et al.*, 1988), where one allele is frequently deleted, and mutation often occurs on the remaining allele. These alterations of the p53 gene have been found in many human cancers, such as those of the colon (Baker *et al.*, 1990; Rodriguez *et al.*, 1990), lung (Takahashi *et al.*, 1989; Chiba *et al.*, 1990), breast (Prosser *et al.*, 1990; Bartek *et al.*, 1990), liver (Fujimori *et al.*, 1991; Slagle *et al.*, 1991) and brain (Nigro *et al.*, 1989). Wild-type p53 product can suppress transformation *in vitro*, whereas mutated p53 protein may inactivate the wild-type p53 function, resulting in cell transformation (Finlay *et al.*, 1989; Eliyahu *et al.*, 1989; Baker *et al.*, 1990). These findings are consistent with the concept that the wild-type p53 gene product functions as a suppressor of neoplastic growth.

Although ovarian cancer is the leading cause of death among all carcinomas of the female reproductive tract, the genetic alterations involved in ovarian carcinoma remain largely unknown. As is the case with other common human carcinomas (Hunter, 1991), accumulation of multiple genetic alterations must be present in ovarian carcinoma, playing significant roles in carcinogenesis and tumour progression. Amplification of the HER-21/*neu* (Slamon *et al.*, 1989), c-*myc* (Zhou *et al.*, 1988) and c-K-*ras* (Filmus & Buick, 1985) genes has been detected in ovarian carcinoma, however the incidence of such amplifications is not high. On the other hand, some data are available on the alterations of p53 gene in ovarian carcinoma (Eccles *et al.*, 1990, 1992; Lee, 1990; Marks *et al.*, 1991; Mazars *et al.*, 1991; Okamoto *et al.*, 1991), but little information is available regarding the prognostic significance of the p53 gene abnormalities.

The present study was undertaken to examine alterations of the p53 gene in a series of 54 ovarian carcinomas using PCR-SSCP[1] analysis (Orita *et al.*, 1989; Hayashi, 1991). The utilisation of DNA samples extracted from paraffin-embedded tissues prompted us to investigate retrospectively allelic losses and mutations of the p53 gene and their association with clinicopathological findings. A chemiluminescent detection system (Beck *et al.*, 1989; Creasey *et al.*, 1991) was adapted for PCR-SSCP analysis in order to eliminate the hazards and long exposure times associated with the original radioactive method. We examined p53 gene mutation not only in exons 5-8, where most of the evolutionarily conserved amino acids are concentrated (Hollstein *et al.*, 1991), but also in exon 4, where some mutations have been reported in ovarian cancer (Okamoto *et al.*, 1991). We also detected allelic losses of p53 gene from a polymorphism in exon 4 (Buchman *et al.*, 1988) represented by PCR-SSCP analysis (Orita *et al.*, 1989; Okamoto *et al.*, 1991).

### Materials and methods

### *Patients*

Fifty-seven patients with ovarian carcinoma were employed in this study. Each patient underwent exploratory laparotomy as part of treatment for ovarian carcinoma at the Gifu University Hospital between 1984 and 1991. Verbal consent to the use of samples for analysis was obtained from patients after full explanation of the nature of the studies to be carried out. Fourteen patients with benign ovarian cyst (seven serous cystadenomas, four mucinous cystadenomas and three dermoid cysts) and three ovaries showing pathologically no abnormalities (as an internal control of constitutional genotype) were also examined. Histological type and grade of the tumours were assigned according to the WHO criteria (Serov & Scully, 1973). The clinical stage was evaluated according to the typing system of the International Federation of Gynecology and Obstetrics (FIGO, 1982). In 3 of the 57 ovarian cancers, PCR-SSCP analyses were unsuccessful because of the poor quality of the DNA extracted. Among the remaining 54 ovarian carcinomas with available data on p53 gene alteration, 51 were of epithelial origin: 19 serous cystadenocarcinomas, 12 clear cell carcinomas, 12 endometrioid adenocarcinomas, seven mucinous cystadenocarcinomas and one undifferentiated carcinoma. The other three were squamous cell carcinomas arising from mature cystic teratoma. The histological grades of those 54 ovarian cancers were: well differentiated in 14 cases, moderately differentiated in 19 cases and poorly differentiated in 21 cases. There were 15 cases in stage I, 11

Correspondence: K. Niwa.
Received 28 February 1994; and in revised form 26 July 1994.

in stage II, 20 in stage III and eight in stage IV. None of the 54 patients with ovarian carcinomas had received any chemotherapy or radiotherapy prior to the surgery. All of them had received cisplatin/doxorubicin/cyclophosphamide chemotherapy following primary surgical exploration.

*PCR–SSCP analysis*

All of the DNA samples were extracted from tumour tissues embedded in paraffin blocks. Areas of tumours containing a large proportion of neoplastic cells (approximately over 90%) were identified histopathologically in tissue sections. The serial 20–30 tissue sections (20 $\mu$m thick) with abundant tumour cells were deparaffined in xylene and incubated for 48–96 h at 37°C in lysis buffer containing 0.1 mg $ml^{-1}$ proteinase K (Jackson *et al.*, 1991). After complete digestion, DNA was purified by deproteination with saturated sodium chloride (Miller *et al.*, 1988) and precipitation by ethanol. The concentration of DNA was spectrophotometrically determined, and the DNA solution at a concentration of 100 ng $ml^{-1}$ was used as a template for the following PCR procedure.

To amplify coding exons 4–8 of the p53 gene, the following oligonucleotide primers were designed using published sequence data (Buchman *et al.*, 1988) and synthesized by the phosphoramidite method: E4U, 5′-TTCACCCATCTACAG-TCC-3′; E4D, 5′-CTCAGGGCAACTGACCGT-3′; E5U, 5′-TTCCTCTTCCTGCAGTACTCC-3′; E5D, 5′-GCCCCAG-CTGCTCACCATCGC-3′; E6U, 5′-CACTGATTGCTCTT-AGGTC-3′; E6D, 5′-AGTTGCAAACCAGACCTCA-3′; E7U, 5′-TCCTAGGTTGGCTCTGAC-3′; E7D, 5′-CAAGT-GGCTCCTGACCTG-3′;E8U, 5′-CCTATCCTGAGTAGT-GGTAA-3′; E8D, 5′-CCTGCTTGCTTACCTCGCT-3′. For instance, E4U and E4D are upstream (sense) and downstream (antisense) primers, respectively, covering exon 4, the expected sizes of PCR fragments are 309 bp (exon 4), 214 bp (exon 5), 141 bp (exon 6), 131 bp (exon 7) and 162 bp (exon 8). For chemiluminescent detection of SSCP, all of the primers were biotinylated at the 5′ terminus with the Biotin-ON phosphoramidite (Clontech, Palo Alto, CA, USA). The PCR mixture in a total volume of 20 $\mu$l contained 100 ng of genomic DNA, 0.2 mM each of dNTPs, 0.25 mM each of biotinylated primers, 1.5 mM magnesium chloride, 50 mM potassium chloride, 10 mM Tris–HCl, pH 8.3, 0.01% gelatin and 0.5 units of *Taq* DNA polymerase. Thirty cycles consisting of 20 s at 94°C, 20 s at 55°C and 30 s at 72°C were performed.

For SSCP analysis, a 2 $\mu$l aliquot of the PCR product was diluted 50-fold with a loading solution containing 20 mM EDTA, pH 8.0, 95% formamide, 0.05% bromophenol blue and 0.05% xylene cyanol. The diluted samples were heated at 90°C for 5 min, and 2 $\mu$l of the samples were applied to two non-denaturing polyacrylamide gels (0.5 × HydroLink MDE gel; AT Biochem, Malvern, PA, USA), one containing 5% glycerol and the other without glycerol. Electrophoresis in the gels with and without glycerol was performed at a constant power of 6 W for 12 h and 6 h respectively, at room temperature. Then, the DNA fragments in the gels were transferred to a nylon membrane (Immobilon-S; Millipore, Bedford, MA, USA) by capillary blotting for 30 min using 0.5 × Tris-borate buffer without any pretreatment of the gels. By this procedure, about a third of the DNA fragments was transferred to the membrane surface. The gels containing untransferred DNA fragments were stored at 37°C in humidified air until cutting of the gel pieces containing a mobility-shift band. After transfer, the membrane was dried, and the DNA fragments were cross-linked to the membrane by ultraviolet irradiation (33,000 mJ $cm^{-2}$ at 254 nm). The bands of biotin-labelled DNA fragments were detected with a Plex 5 Chemiluminescent Subkit (New England BioLabs, Beverly, MA, USA) basically according to the report of Creasey *et al.* (1991). The luminescing band patterns were recorded on standard X-ray film with an exposure time of 15–30 min. In the SSCP analysis for exon 4, the signal intensity of the bands was determined by a Bio Image 505 System (Millipore).

*Sequencing analysis*

By laying the developed film of SSCP analysis on the stored gel, an area of the gel containing a mobility-shift band was determined and cut out. DNA fragments were eluted from excised gel slices by the crush and soak method (Sambrook *et al.*, 1989), and were amplified by a PCR under the same conditions as described above. The sequences of the primers used were identical to those used in the prior PCR but were not biotinylated. The PCR products were fractionated by electrophoresis through 12% non-denaturing polyacrylamide gels. The DNA fragments with an expected size were eluted from excised gel slices, and ligated to pT7 Blue T-vector (Novagen, Madison, WI, USA). After transformation of Nova Blue competent cells (Novagen), double-strand plasmid DNA was isolated, and both sense and antisense strands were sequenced by using a Circum Vent thermal cycle dideoxy DNA sequencing kit (New England BioLabs) with biotinylated M13/pUC reverse sequencing primer and biotinylated T7 promoter primer. Sequencing products were electrophoresed on denaturing polyacrylamide gels (5% HydroLink Long Ranger gel; AT Biochem) at a constant power of 75 W for 3 h. The procedure for chemiluminescent detection of DNA band patterns was identical to that in SSCP analysis. Sequence analysis was carried out on several clones from independent PCR products to eliminate sequencing errors due to polymerase misincorporations. In addition, it was confirmed that the PCR fragment generated from the cloned DNA using the same set of primers migrated to the same position as the abnormal fragment generated from the respective cellular DNAs.

*Statistics*

Statistical analyses were done using Fisher's exact probability test or Student's *t*-test. Survival estimates were calculated using the product limit method of Kaplan and Meier (1956). Differences in survival were tested using the log-rank statistic (Mantel, 1966).

### Results

*Allelic losses of p53 gene*

Since there is sequence polymorphism at codon 72 in exon 4 (Buchman *et al.*, 1988), the DNA fragment covering exon 4 was amplified by a PCR, and analysed by the SSCP method. Depending on the presence or absence of 5% glycerol in the gel, the mobilities and the separation patterns of allelic bands varied but they were evident and reproducible under each condition. Figure 1 shows the representative results of SSCP analysis of the gel without glycerol. Polymorphism at codon 72 in exon 4 was represented by three bands with different mobility. Sequence analysis revealed that the band on the top (band 1, B1) and the band in the middle (band 2, B2) contained the arginine codon (CGC) and the proline codon (CCC), respectively, and that the band at the bottom corresponded to each of the complementary strands which comigrated under these conditions. Allelic loss was able to be detected by PCR–SSCP analysis without corresponding normal tissues, because a residual weak signal, presumably derived from normal cells in the tumour specimen, was observed in most of the tumours with a heterozygous genotype (for example cases 2, 3, 4, 7 and 8 in Figure 1). According to the criteria of Okamoto *et al.* (1991), allelic loss is considered to have occurred if tumours show a heterozygous genotype and the signal intensity of a paired allelic fragment is less than 40% of the other paired fragments.

Signal intensity of one of the bands B1 and B2 was reduced in 18 of the 26 ovarian carcinomas with a heterozygous genotype, indicating the presence of allelic loss (for example cases 2, 3, 4 and 8 in Figure 1). Moreover, in six cases with a homozygous genotype and p53 gene mutation (cases 1, 5, 6, 9, 12 and 14 in Figure 1 and Table I), allelic loss was confirmed by a reduced intensity of the corresponding normal bands as described below. The 32 cases of the 54 ovarian carcinomas examined were informative, and allelic loss was detected in any of the seven informative cases with benign ovarian cyst (walls of three serous cystadenomas, two mucinous cystadenomas and two dermoid cysts).

*Mutations of p53 gene*

Since allelic loss of p53 gene was frequently observed in ovarian carcinomas, we further examined for mutations of

**Figure 1** SSCP analysis of PCR fragments of exon 4 of the p53 gene. Electrophoresis was performed using non-denaturing polyacrylamide gel without glycerol at room temperature. Numbers on the top of each lane correspond to case numbers shown in Table 1. Lane N was loaded with PCR fragments generated from normal human placental DNA. The bands B1 and B2 correspond to the allele carrying CGC and that carrying CCC, respectively, at codon 72. Cases 1, 5 and 6 were found to be constitutionally homozygous. The ratio of signal intensity of bands B1 and B2 (a weaker signal to the other one) was: lane N, 0.881; lane 2, 0.273; lane 3, 0.182; lane 4, 0.235; lane 7, 0.702; and lane 8, 0.342.

the gene by PCR-SSCP analysis. The five DNA segments covering exons 4, 5, 6, 7 and 8 were amplified by a PCR and analysed by the SSCP method. Figure 2 shows the representative results of SSCP analysis of the gel without glycerol. The PCR fragments of normal placental DNA exhibited two bands for exons 6 and 8, representing the two complementary single strands of DNA. On the other hand, in the cases of exons 5 and 7, the two slower moving bands (B1 and B2) and the two faster moving bands (B3 and B4) were observed for normal placental DNA. The DNAs in bands B1 and B2 were confirmed to have exactly the same nucleotide sequence, suggesting that the strands having the same sequence can have two stable conformations. The DNAs in bands B3 and B4 also had the same nucleotide sequence. These results indicate that the single-stranded DNA in bands B1 and B2 is complementary to that in bands B3 and B4.

In the SSCP analysis of the gel without glycerol, 14 ovarian carcinomas showed the bands with different mobility in one of the three PCR fragments (exons 5, 6 and 7 in Figure 2), whereas none of the walls of 14 benign ovarian cysts and three normal ovaries examined showed any band with different mobility. SSCP analysis of the gel containing 5% glycerol yielded the same result, although the pattern of separations of the bands was varied. When a normal allele is retained in the tumour, the intensity of bands for mutated allele should be the same as or less than that of bands for normal allele. In cases 1, 2, 4, 5, 6, 8, 9, 10, 11, 12 and 14 in Figure 2, the intensity of bands with different mobility was higher than that of the corresponding normal bands, indicating the presence of loss of normal allele. However, in cases 3, 7 and 13 in Figure 2, we were not able to detect any loss of normal allele, because the intensity of the band with different mobility was lower than that of the bands for normal allele.

In the 14 DNA samples showing bands with different mobility, sequence analyses were performed to confirm the presence of mutated p53 genes and to determine the type of mutations (Figure 3). As summarised in Table I, sites of mutations were distributed between codons 143 and 249, mutations tended to cluster in exons 5 and 7 [9 of 14 in exon 7 (64%); 4 in exon 5 (29%); and 1 in exon 6 (7%)]; 11 (79%) of 14 mutations were detected on the highly conserved regions (Hollstein *et al.*, 1991), and the other three mutations were found at codons corresponding to amino acids conserved among several species (Hollstein *et al.*, 1991). Thirteen of the 14 cases with mutation had concomitant loss of the normal allele. Twelve mutations revealed single nucleotide substitutions (missense mutations), ten of which were transitions (from G:C to A:T in eight cases and from A:T to G:C in one case), and the other two were transversions from G to T. Cases 4 and 6 revealed a three-base deletion, resulting in the deletion of codons 218 (valine) and 234 (tyrosine) respec-

**Table I** Alteration of the p53 gene and correlation of allelic loss in ovarian carcinomas

| Case | *Histology* *Type*[a] | *Grade*[b] | *Allelic loss*[c] | *Exon* | *Codon* | *Mutation* *Nucleotide change* | *Amino acid change* | *FIGO stage* |
|---|---|---|---|---|---|---|---|---|
| 1 | SCA | W | +, CH, LON | 5 | 181[d] | CGC to CAC[e] | Arg to His | I |
| 2 | SCA | P | +, LOB2, LON | 7 | 245[d] | GGC to AGC | Gly to Ser | II |
| 3 | SCA | P | +, LOB1 | 7 | 248[d] | CGG to CAG[e] | Arg to Gln | III |
| 4 | SCA | M | +, LOB2, LON | 6 | 218 | GTG deleted | Val deleted | III |
| 5 | SCA | P | +, CH, LON | 7 | 245[d] | GGC to GTC | Gly to Val | III |
| 6 | SCA | M | +, CH, LON | 7 | 234[d] | TAC deleted | Tyr deleted | IV |
| 7 | SCA | P | −, ROH | 7 | 249[d] | AGG to AGT | Arg to Ser | IV |
| 8 | SCA | P | +, LOB1, LON | 7 | 249[d] | AGG to AGT | Arg to Ser | IV |
| 9 | EA | W | +, CH, LON | 5 | 175[d] | CGC to CAC[e] | Arg to His | III |
| 10 | EA | M | +, LOB2, LON | 7 | 245[d] | GGC to AGC | Gly to Ser | III |
| 11 | EA | M | +, LOB1, LON | 5 | 163 | TAC to TGC | Tyr to Cys | III |
| 12 | EA | M | +, CH, LON | 7 | 241[d] | TCC to TTC | Ser to Phe | III |
| 13 | EA | M | +, LOB2 | 5 | 143 | GTG to ATG | Val to Met | IV |
| 14 | MCA | W | +, CH, LON | 7 | 248[d] | CGG to TGG[e] | Arg to Trp | I |

[a]SCA, serous cystadenocarcinoma; EA, endometrioid adenocarcinoma; MCA, mucinous cystadenocarcinoma. [b]W, well differentiated; M, moderately differentiated; P, poorly differentiated. [c]CH, constitutional homozygosity; LOB1, loss of band B1; LOB2, loss of band B2; ROH, retention of heterozygosity; LON, loss of normal allele. [d]Codons within evolutionary conserved regions of the p53 gene. [e]Transitions at a CpG dinucleotide.

tively. Transitions at a CpG dinucleotide were detected in four of the 14 mutations.

*Clinicopathological features and prognosis of ovarian carcinomas showing p53 gene alteration*

The heterogeneity of ovarian carcinomas examined with respect to the presence of p53 gene alterations prompted us to analyse possible correlations with clinicopathological characteristics of ovarian carcinomas. As summarised in Table II, there was no significant relationship between p53 gene alterations and age at diagnosis, clinical stage or histological grade. The rate of p53 gene abnormalities in ovarian carcinomas tended to be increased in advanced stage (over FIGO stage II). However, no significant relationship was also found between allelic loss and histological type.

On the other hand, mutations were frequent in serous cystadenocarcinoma (42%) and endometrioid adenocarcinoma (42%) but infrequent in mucinous cystadenocarcinoma (14%). The nucleotide changes in serous cystadenocarcinoma did not show any specific pattern: three

**Figure 2** Detection of p53 gene mutations by SSCP analysis. Electrophoresis was performed using non-denaturing polyacrylamide gel without glycerol at room temperature. Numbers on the top of each lane correspond to case numbers shown in Table I. Lane N was loaded with PCR fragments generated from normal human placental DNA. Normal alleles were indicated by the bands B1, B2, B3 and B4 in exons 5 and 7, and by arrowheads in exons 6 and 8. Abnormal shifted bands were detected in cases 1–14.

**Figure 3** Sequencing analysis of p53 gene mutations. Case numbers correspond to those shown in Table I. *Upper right*: A point mutation at codon 245 (exon 7) from GGC to AGC. *Middle right*: A deletion of three bases (TAC) at codon 234 (exon 7). *Lower right*: a point mutation at codon 163 (exon 5) from TAC to TGC. The arrows indicate the positions of mutations or deletions. The wild-type sequences are shown on the left.

**Table II** Correlation between p53 gene alteration and clinicopathological parameters in ovarian carcinoma

| | *Allelic loss*[a] | | *Mutation*[b] | |
|---|---|---|---|---|
| | *Positive* | *Negative* | *Positive* | *Negative* |
| Median age at diagnosis | 55.0 | 50.5 | 49.5 | 51.0 |
| FIGO stage | | | | |
| I | 5 (56%)[c] | 4 (44%) | 2 (13%) | 13 (87%) |
| II | 6 (86%) | 1 (14%) | 1 (9%) | 10 (91%) |
| III | 10 (83%) | 2 (17%) | 7 (35%) | 13 (65%) |
| IV | 3 (75%) | 1 (25%) | 4 (50%) | 4 (50%) |
| II–IV | 19 (83%) | 4 (17%) | 12 (31%) | 27 (69%) |
| Histological grade | | | | |
| Well differeniated | 4 (57%) | 3 (43%) | 3 (21%) | 11 (79%) |
| Moderately differentiated | 8 (62%) | 5 (38%) | 7 (37%) | 12 (63%) |
| Poorly differentiated | 10 (83%) | 2 (17%) | 4 (19%) | 17 (81%) |
| Histological type[c] | | | | |
| SCA | 9 (82%) | 2 (18%) | 8 (42%)[e] | 11 (58%) |
| CCC | 4 (67%) | 2 (33%) | 0 (0%) | 12 (100%) |
| EA | 6 (75%) | 2 (25%) | 5 (42%)[e] | 7 (58%) |
| MCA | 2 (50%) | 2 (50%) | 1 (14%) | 7 (86%) |
| SCC | 2 (100%) | 0 (0%) | 0 (0%) | 3 (100%) |
| UC | 1 (100%) | 0 (0%) | 0 (0%) | 1 (100%) |

[a]32 patients with available data on allelic loss of p53 gene. [b]54 patients with available data on mutation of p53 gene. [c]Number of patients (%). [d]SCA, serous cystadenocarcinoma; CCC, clear cell carcinoma; EA, endometrioid adenocarcinoma; MCA, mucinous cystadenocarcinoma; SCC, squamous cell carcinoma; UC, undifferentiated carcinoma. [e]Significantly higher than the group of clear cell carcinoma ($P < 0.05$).

**Table III** Relation between p53 gene alteration and clinical parameters in ovarian serous tumours and endometroid adenocarcinoma

| | *Number of cases examined* | *Number of cases in p53 mutation 'positive'* |
|---|---|---|
| Pathology | | |
| Serous tumours | | |
| Serous cystadenoma | 7 | 0 (0%) |
| Serous cystadenocarcinoma | | |
| Well differentiated | 3 | 1 (33%) |
| Moderately differentiated | 6 | 2 (33%) |
| Poorly differentiated | 10 | 5 (50%) |
| FIGO stage | | |
| I | 3 | 1 (33%) |
| II | 2 | 1 (50%) |
| III | 9 | 3 (33%) |
| IV | 5 | 3 (60%) |
| II-IV | 16 | 7 (44%) |
| Endometrioid adenocarcinoma | | |
| Well differentiated | 6 | 1 (17%) |
| Moderately differentiated | 6 | 4 (67%) |
| Poorly differentiated | 0 | 0 |
| FIGO stage | | |
| I | 2 | 0 (0%) |
| II | 2 | 0 (0%) |
| III | 6 | 4 (67%) |
| IV | 2 | 1 (50%) |
| II to IV | 10 | 5 (50%) |

cases of G:C to A:T transition, two cases of G to T transversion, two cases of three-base deletion and one case of A:T to G:C transition. On the other hand, G:C to A:T transitions were the most frequent substitution (4/5 cases) in endometrioid adenocarcinoma. No mutation was found in 12 clear cell carcinomas, three squamous cell carcinomas and one undifferentiated carcinoma.

We were able to evaluate p53 gene alteration in both the primary tumour and 1-3 metastases in three patients with allelic loss and mutation (cases 3, 8 and 12) and four patients with allelic loss but without mutation, in whom the tumour was obtained at the initial cytoreductive surgery. In all seven patients, the genetic alteration revealed in the metastases was identical to that in the primary tumour. We were also able to compare the status of the p53 gene before and after chemotherapy in three patients with allelic loss and mutation (cases 5, 7 and 12) and in three patients with allelic loss but without mutation, in whom the tumour was obtained at the second-look or subsequent laparotomy. The status of the p53 gene did not change during the course of post-operative chemotherapy in any of the six patients.

In Table III, the relation between p53 alteration and either histological grade or FIGO stage in serous tumours and endometrioid adenocarcinomas is shown. In ovarian serous tumours, allelic loss or the mutation of p53 gene was not detected in benign serous adenomas, whereas the incidence of mutation in serous cystadenocarcinoma was relatively high (8/19, 43%) and the incidence of the mutation seemed to be higher in accordance with de-differentiation. The incidence in FIGO III/IV seemed to be slightly higher than that in FIGO I/II. In endometrioid adenocarcinoma, the incidence in moderately differentiated cases seemed to be higher than in well-differentiated cases. According to the FIGO stage, the incidence of p53 mutation in FIGO stage III/IV seemed to be higher than that in FIGO stage I/II.

One (8%) of the 12 evaluable patients with p53 gene mutation (six with progressive disease and six second looks) and four (17%) of the 24 evaluable patients without p53 gene mutation (nine with progressive disease and 15 second looks) revealed a negative second look; no relationship was found between the presence of p53 gene mutation and the incidence of surgically documented complete response ($P>0.4$). Among the five patients who had a negative second-look laparotomy, one patient without p53 gene mutation has

**Figure 4** Relationship between p53 gene mutation and survival in 31 patients with ovarian cancer. ——, survival curve in 13 patients with p53 gene mutation; ----, survival curve in 18 patients without p53 gene mutation. The tick marks represent the follow-up times of living patients.

subsequently showed recurrence of the disease. The other three patients are alive with no evidence of the disease. Since most of the p53 gene mutations confirmed in this study were observed in serous cystadenocarcinoma and endometrioid adenocarcinoma (Table I), we examined the relation between p53 gene mutation and survival time in 31 patients (19 patients with serous cystadenocarcinoma and 12 patients with endometrioid adenocarcinoma) (Figure 4). Although the median survival time of the 13 patients with p53 gene mutation (20.1 months) was somewhat worse than that of the 18 patients without p53 gene mutation (28.2 months), the difference normalised by clinical stage was not statistically significant ($P>0.5$).

### Discussion

Several reports (Okamoto *et al.*, 1991; Mashiyama *et al.*, 1991; Tamura *et al.*, 1991; D'Amico *et al.*, 1992) have documented the effectiveness of PCR-SSCP analysis for detecting mutations of p53 gene. In this study we extracted DNA from paraffin-embedded tissues because PCR does not require high molecular weight DNA as a template. This approach makes possible retrospective analysis of tissue specimens several years old (Jackson *et al.*, 1991), although the length of DNA fragments that can be amplified by PCR depends on the integrity of the template DNA. Shorter DNA fragments are better suited for detection of mutations by SSCP analysis; the sensitivity of SSCP analysis is more than 99% for DNA fragments of 100-300 bp (Hayashi, 1991). When the DNA fragments analysed by SSCP are less than approximately 300 bp, the possibility of a false-negative result is quite low under two conditions (Hayashi, 1991; Hayashi & Yandel, 1993). The SSCP electrophoresis was performed in the gels with and without 5% glycerol in the present study. For these reasons, we performed PCRs on each of exons 4-8 of the p53 gene, resulting in successful SSCP analyses on most of the DNA samples extracted from paraffin-embedded tissues.

Using PCR-SSCP analysis, we detected allelic losses of the p53 gene in 24 (75%) of the 32 informative ovarian cancers, whereas, as expected, allelic loss was not detected in any of the seven benign ovarian cysts with heterozygous genotypes. The frequency of allelic loss in the ovarian cancers examined in this study was similar to that in the previous reports (Eccles *et al.*, 1990; Lee *et al.*, 1990; Okamoto *et al.*, 1991) on loss of heterozygosity of chromosome 17 detected by restriction fragment length polymorphism analysis. We also found 14 cases with p53 gene mutation (26%) in 54 ovarian cancers. This frequency of mutation was lower than that of allelic loss, and not as high as those for carcinomas of the colon (Baker *et al.*, 1990; Rodriguez *et al.*, 1990) and lung (Takahashi et al., 1989; Chiba et al., 1990). We regard our value as a conservative estimate for the following reasons. All primers were designed such that they included several of the first or last nucleotides of exons (Buchman et al., 1988). Therefore, mutations occurring in the regions included in the primers, such as splice site mutation, could not be detected. It is also possible that mutations might be present in a region of p53 gene that was not targeted in the present study. Furthermore, there may be undetectable mutations by SSCP analysis, because the strands with different sequences sometimes have the same stable conformation and co-migrate, as in the case of exon 4 (Figure 1). We also cannot rule out the possibility that genetic mutations are masked by a high proportion of non-cancerous cells contained in tumour specimens.

Sequence analysis revealed 12 single point mutations and two deletions. Nine cases had not only p53 gene mutations in the highly conserved regions of p53 gene, but also allelic losses of p53 gene, strongly suggesting the complete loss of normal p53 function. Most of the p53 gene mutations confirmed in this study were observed in serous cystadenocarcinoma and endometrioid adenocarcinoma. In serous tumours, no alterations of p53 gene could be detected in benign serous cystadenomas. From the point of view of relationship between differentiation and p53 alteration in serous tumours, the incidence of the alteration seemed to be increased in accordance with de-differentiation. In endometrioid adenocarcinoma, a similar tendency was also found. The nucleotide changes were not confined to a specific pattern in serous cystadenocarcinoma. In contrast, G:C to A:T transitions constitute the majority of mutations in endometrioid adenocarinoma. The pattern of mutations seen in the present study is similar to that reported for other epithelial tumours, and the frequency of the G:C to A:T transversion in ovarian endometrioid adenocarcinoma is considered to resemble to that in hepatocellular carcinomas (Caron de Fromentel & Soussi, 1992). No mutation was detected in 12 clear cell carcinomas, in agreement with the result of Okamoto et al. (1991). It is probable that the disparity between these mutational spectra is due to differences in metabolic and DNA repair capacities among different cell types (Harris, 1989). One of the features of the p53 mutational spectra in human cancers is that transitions at CpG dinucleotides contribute heavily to the mutational frequency in many cancers (Hollstein et al., 1991). In the present series of ovarian cancer, transitions at CpG sites were found in four (29%) of 14 mutations, and this frequency was relatively low compared with that of colon cancer (67%) (27). Methylation of CpG sites and the level of spontaneous deamination may differ in various tissue types.

Eccles et al. (1992) reported the relation between immunohistochemical overexpression of p53 protein in frozen sections and allele loss at 17p in ovarian carcinoma. Their hypothesis using immunohistochemistry could be confirmed by the SSCP and direct sequencing in the present study. In particular, in ovarian serous tumorigenesis in the present study, allelic loss or mutation of p53 gene was not detected in benign serous adenoma, whereas the incidence of alteration in serous cystadenocarcinoma was relatively high (43%) and the incidence of the alteration seemed to be higher in accordance with de-differentiation. Recent work on colorectal tumours (Baker et al., 1990b) suggested that alteration of the p53 gene might be the rate-limiting step in tumorigenesis. Thus, in ovarian serous tumorigenesis, the point mutation in the p53 gene might be the rate-limiting step and the loss of the remaining wild-type allele might occur afterwards as a tumour changes from benign to malignant.

In this study, we determined whether p53 gene mutation correlated with prognostic factors in ovarian cancer. As a result, no correlation was revealed between p53 gene mutation and histological grade, which has been used with some success for predicting the eventual clinical outcome (Ozols et al., 1992). We also found no relationship between p53 gene mutation and response to primary therapy. No correlation was found between p53 gene mutation and survival time in the 31 patients with serous cystadenocarcinoma or endometrioid adenocarcinoma, with or without cases with the mutations identified.

The present study suggests that alterations of the p53 gene might be involved in the development of a proportion of ovarian carcinomas, especially in serous tumorigenesis, and might play an important role in commencement of invasion. However, they could not have an important role as a mechanism for aggressive biological behaviour of the disease. Further investigations will be required to elucidate the multiple genes, oncogenes and tumour-suppressor genes involved in the ovarian carcinogenesis and to understand their biological and clinical significance.

### References

BAKER, S.J., MARKOWITZ, S., FEARON, E.R., WILLSON, J.K.W. & VOGELSTEIN, B. (1990a). Suppression of human colorectal carcinoma cell growth by wild-type p53. *Science*, **249**, 912–915.

BAKER, J.S., PREISINGER, A.C., JESSUP, J.M., PARASKEVA, C., MARKOWITZ, S., WILSON, J.K.V., HAMILTON, S. & VOGELSTEIN, B. (1990b). p53 gene mutations occur in combination with 17p allelic deletions as late events in colorectal tumorigenesis. *Cancer Res.*, **50**, 7717–7722.

BARTEK, J., IGGO, R., GANNON, J. & LANE, D.P. (1990). Genetic and immunochemical analysis of mutant p53 in human breast cancer cell lines. *Oncogene*, **5**, 893–899.

BECK, S., O'KEEFE, T., COULL, M. & KÖSTER, H. (1989). Chemiluminescent detection of DNA: application for DNA sequencing and hybridization. *Nucleic Acid Res.*, **17**, 5115–5123.

BUCHMAN, V.L., CHUMAKOV, P.M., NINKINA, N.N., SAMARINA, O.P. & GEORGIEV, G.P. (1988). A variation in the structure of the protein-coding region of the human p53 gene. *Gene*, **701**, 245–252.

CARON DE FROMENTEL, C. & SOUSSI, T. (1992). TP53 tumor suppressor gene: a model for investigating human mutagenesis. *Genes Chrom. Cancer*, **4**, 1–15.

CREASEY, A., D'ANGIO, L. Jr, DUNNE, T.S., KISSINGER, C. O'KEEFE, T., PERRY-O'KEEFE, H., MORAN, L.S., ROSKEY, M., SCHILDKRAUT, I., SEARS, L.E. & SLATKO, B. (1991). Application of a novel chemiluninescence-based DNA detection method to single-vector and multiplex DNA sequencing. *BioTechniques*, **11**, 102–109.

CHIBA, I., TAKAHASHI, T., NAU, M.M., D'AMICO, D., CURIEL, D.T., MITSUDOMI, T., BUCHHAGEN, D.L., CARBONE, D., PIANNTADOSI, S. & KOGA, H. (1990). Mutations in the p53 gene are frequent in primary, resected non-small cell lung cancer. *Oncogene*, **5**, 1603–1610.

D'AMICO, D., CARBONE, D., MITSUDOMI, T., NAU, M., FEDORKO, J., RUSSELL, E., JOHNSON, B., BUCHHAGEN, D., BODNER, S., PHELPS, R., GAZDAR, A. & MINNA, J.D. (1992). High frequency of somatically acquired p53 mutations in small-cell lung cancer cell lines and tumors. *Oncogene*, **7**, 339–346.

ECCLES, D.M., CRANSTON, G., STEEL, C.M., NAKAMURA, Y. & LEONARD, R.C.F. (1990). Allele losses on chromosome 17 in human epithelial ovarian carcinoma. *Oncogene*, **5**, 1599–1601.

EHYAHU, D., MICHALOVITZ, D., ELIYAHU, S., PINHASI-KIMHI, O. & OREN, M. (1989). Wild-type p53 can inhibit oncogene-mediated focus formation. *Proc. Natl Acad. Sci. USA*. **86**, 8763–8767.

FIGO (1982). *Annual report on the results of treatment in gynecological cancer*, Vol. 18, Kottmeier, H.L. (ed.). International Federation of Gynecologists and Obstetricians, Stockholm.

FINLAY, C.A., HINDS, P.W. & LEVINE, A.J. (1989). The p53 protooncogene can act as a suppressor of transformation. *Cell*, **57**, 1083–1093.

FILMUS, J.E. & BUICK, R.N. (1986). Stability of c-k-*ras* amplification during progression in a patient with adenocarcinoma of the ovary. *Cancer Res.*, **45**, 4468–4472.

FUJIMORI, M., TOKINO, T., HINO, O., KITAGAWA, T., IMAMURA, T., OKAMOTO, E., MITSUNOBU, M., ISHIKAWA, T., NAKAGAWA, H., HARADA, H., YAGURA, M., MATSUBARA, K. & NAKAMURA, Y. (1991). Allelotype study of primary hepatocellular carcinoma. *Cancer Res.*, **51**, 89-93.

HARRIS, C.C. (1989). Interindividual variation among humans in carcinogen metabolism, DNA adduct formation and DNA repair. *Carcinogenesis*, **10**, 1563-1566.

HAYASHI, K. (1991). PCR-SSCP: a simple and sensitive method for detection of mutations in the genomic DNA. *PCR Methods Appl.*, **1**, 34-38.

HAYASHI, K. & YANDELL, D.W. (1993). How sensitive is PCR-SSCP? *Hum. Mutat.*, **2**, 338-346.

HOLLSTEIN, M., SIDRANSKY, D., VOGELSTEIN, B. & HARRIS, C.C. (1991). p53 mutations in human cancers. *Science*, **253**, 49-53.

HUNTER, T. (1991). Cooperation between oncogenes. *Cell*, **64**, 249-270.

JACKSON, D.P., HAYDEN, J.D. & QUIRKE, P. (1991). Extraction of nucleic acid from fresh and archival material. In *PCR, A Practical Approach*, McPherson, M.J., Quirke, P. & Taylor G.R. (eds), pp. 29-50. Oxford University Press: Oxford.

KAPLAN, E.L. & MEIER, P. (1956). Nonparametric estimation from incomplete observations. *J. Am. Stat. Assoc.*, **53**, 457-481.

LEE, J.H., KAVANAGH, J.J., WILDRICK, D.M., WHARTON, J.T. & BLICK, M. (1990). Frequent loss of heterozygosity on chromosomes 6q, 11 and 17 in human ovarian carcinomas. *Cancer Res.*, **50**, 2724-2728.

MANTEL, N. (1966). Evaluation of survival data and two new rank order statistics arising in its consideration. *Cancer Chemother. Rep.*, **50**, 163-170.

MARKS, J.R., DAVIDOFF, A.M., KERNS, B.J., HUMPHREY, P.A., PENCE, J.C., DODGE, R.K., CLARKE-PEARSON, D.L. IGLEHART, J.D., BAST, R.C. Jr & BERCHUCK, A. (1991). Overexpression and mutation of p53 in epithelial ovarian cancer. *Cancer Res.*, **51**, 2979-2984.

MASHIYAMA, S., MURAKAMI, Y., YOSHIMOTO, T., SEKIYA, T. & HAYASHI, K. (1991). Detection of p53 gene mutations in human brain tumors by single-strand conformation polymorphism analysis of polymerase chain reaction products. *Oncogene*, **6**, 1313-1318.

MAZARS, R., PUJOL, P., MAUDELONDE, T., JEANTEUR, P. & THEILLET, C. (1991). p53 mutations in ovarian cancer: a late event? *Oncogene*, **6**, 1685-1690.

MILLER, S.A., DYKES, D.D. & POLESKY, H.F. (1988). A simple salting out procedure for extracting DNA from human nucleated cells. *Nucleic Acids Res.*, **16**, 1215.

NIGRO, J.M., BAKER, S.J., PREISINGER, A.C., JESSUP, J.M., HOSTETTER, R., CLEARY, K., BIGNER, S.H., DAVIDSON, N., BAYLIN, S., DEVILEE, P., GLOVER, T., COLLINS, F.S., WESTON, A., MODALI, R., HARRIS, C.C. & VOGELSTEIN, B. (1989). Mutations in the p53 gene occur in diverse human tumour types. *Nature*, **342**, 705-708.

OKAMOTO, A., SAMESHIMA, Y., YOKOYAMA, S., TERASHIMA, Y., SUGIMURA, T., TERADA, M. & YOKOTA, J. (1991). Frequent allelic losses and mutations of the p53 gene in human ovarian cancer. *Cancer Res.*, **51**, 5171-5176.

ORITA, M., SUZUKI, Y., SEKIYA, T. & HAYASHI, K. (1989). Rapid and sensitive detection of point mutations and DNA polymorphisms using the polymerase chain reaction. *Genomics*, **5**, 874-879.

OZOLS, R.F., RUBIN, S.C., DEMBO, A.J. & ROBBOY, S.J. (1992). Epithelial ovarian cancer. In Principles and Practice of Gynecologic Oncology, Hoskins, W.J., Perez, C.A. & Young, R.C. (eds), pp. 731-782. J.B. Lippincott: Philadelphia.

PROSSER, J., THOMPSON, A.M., CRANSTON, G. & EVANS, H.J. (1993). Evidence that p53 behaves as a tumor suppressor gene in sporadic breast tumors. *Oncogene*, **5**, 1573-1579.

RODRIGUEZ, N.R., ROWAN, A., SMITH, M.E.F., KERR, I.B., BODMER, W.F., GANNON, J.V. & LANE, D.P. (1990). p53 mutations in colorectal cancer. *Proc. Natl Acad. Sci. USA*, **87**, 7555-7559.

SAMBROOK, J., FRITSCH, E.F. & MANIATIS, T. (1989). In *Molecular Cloning: A Laboratory Manual*, Vol. 1, Sambrook, J., Fritsch, E.F. & Maniatis, T. (eds), pp. 6.1-6.62. Cold Spring Harbor Laboratory Press: Cold Spring Harbor, NY.

SEROV, S.F. & SCULLY, R.E. Histological typing of ovarian tumors. In *International Histological Classification of Tumors*, No. 9 World Health Organization: Geneva.

SLAGLE, B., ZHOU, Y.-Z. & BUTEL, J.S. (1991). Hepatitis B virus integration event in human chromosome 17p near the p53 gene identifies the region of the chromosome commonly deleted in virus-positive hepatocellular carcinomas. *Cancer Res.*, **51**, 49-54.

SLAMON, D.J., GODOLPHIN, W., JONES, L.A., HOLT, J.A., WONG, S.G., KEITH, D.E., LEVIN, W.J., STUART, S.G., UDOVE, J., ULLRICH, A. & PRESS, M.F. (1989). Studies of the HER-2/*neu* proto-oncogene in human breast and ovarian cancer. *Science*, **244**, 707-712.

TAKAHASHI, T., NAU, M.M., CHIBA, I., BIRRER, M.J., ROSENBERG, R.K., VINOCOUR, M., LEVITT, M., PASS, H., GAZDAR, A.F. & MINNA, K.J.D. (1989). p53: a frequent target for genetic abnormalities in lung cancer. *Science*, **246**, 4912-4914.

TAMURA, G., KIHANA, T., NOMURA, K., TERADA, M., SUGIMURA, T. & HIROHASHI, S. (1991). Detection of frequent p53 gene mutations in primary gastric cancer by cell sorting and polymerase chain reaction single-strand conformation polymorphism analysis. *Cancer Res.*, **51**, 3056-3058.

UMESH, M., WOLF, D. & FROSSARD, P.M. (1988). *Ban* II and *Sca* I RFLPs at the human p53 gene locus. *Nucleic Acids Res.*, **16**, 7757.

ZHOU, D.J., GONZALEZ-CADAVID, N., AHUJA, H., BATTIFORA, H., MOORE, G.E. & CLINE, M.J. (1988). A unique pattern of proto-oncogene abnormalities in ovarian adenocarcinomas. *Cancer*, **62**, 1573-1576.